# Preparation, Characterization and Stability of Calcium-Binding Peptides Derived from Chicken Blood

**DOI:** 10.3390/foods13152368

**Published:** 2024-07-26

**Authors:** Jing Yang, Jing Shi, Ying Zhou, Ye Zou, Weimin Xu, Xiudong Xia, Daoying Wang

**Affiliations:** 1 Jiangsu Key Laboratory for Food Quality and Safety-State Key Laboratory Cultivation Base, Ministry of Science and Technology, Nanjing 210014, China; 20210007@jaas.ac.cn; 2 Institute of Agri-Products Processing, Jiangsu Academy of Agricultural Sciences, Nanjing 210014, China; yuyuss29@163.com (J.S.); 20180003@jaas.ac.cn (Y.Z.); 19910015@jaas.ac.cn (W.X.); 3 College of Food Science, Xizang University of Agriculture and Animal Husbandry, Nyingchi 860000, China; 17689549021@163.com

**Keywords:** chicken hemoglobin peptides, calcium-binding capacity, characterization, stability

## Abstract

Calcium-binding peptides have gained significant attention due to their potential applications in various fields. In this study, we aimed to prepare, characterize, and evaluate the stability of calcium-binding peptides derived from chicken blood. Chicken hemoglobin peptides (CPs) were obtained by protease hydrolysis and were applied to prepare chicken hemoglobin peptide–calcium chelate (CP-Ca). The preparation conditions were optimized, and the characteristics and stability of CP-Ca were analyzed. The optimal chelating conditions were determined by single-factor and response surface tests, and the maximum calcium ion chelating rate was 77.54%. Amino acid analysis indicated that glutamic acid and aspartic acid motifs played an important role in the chelation of the calcium ions and CP. According to the characterization analysis, CP-Ca was a different substance compared with CP; calcium ions chelated CPs via the sites of carbonyl oxygen, carboxyl oxygen, and amino nitrogen groups; and after the chelation, the structure changed from a smooth homogeneous plate to compact granular. The stability analysis showed that CP-Ca was stable at different temperatures, pH, and gastrointestinal conditions. The study indicates that chicken blood is a promising source of peptide–calcium chelates, providing a theoretical basis for application in functional foods and improving the utilization value of chicken blood.

## 1. Introduction

Calcium is the most abundant mineral in humans. It plays an essential role in both physiological and biological functions. Calcium deficiency can lead to osteoporosis and rickets and can be alleviated using calcium supplements [1]. Calcium supplements include inorganic calcium, organic calcium, amino acid–calcium chelate, and peptide–calcium chelate. Inorganic calcium has low bioavailability because it is easily affected by phytates, oxalate, and other dietary factors, and organic calcium has toxic side effects [2]. Amino acid-chelates and calcium-binding peptides could overcome these disadvantages because both are safer and more effective than inorganic calcium and organic calcium [3,4]. However, the required amino acids are in a certain proportion, and long-term intake of amino acid-chelated calcium may lead to an imbalance in nitrogen content in the body. Thus, the study of calcium-binding peptides attracts more attention.

In recent years, many researchers have reported producing calcium-binding peptides derived from food protein. Some of the peptides were discovered and characterized from desalted duck eggs [5], antler bone [6], whey protein [7], and egg white peptides [8]. It was reported that calcium-binding peptides were prepared with high bioavailability, absorption, and stability [9]. The chelating activity of peptides has been studied in *Grifola frondosa* protein hydrolysates [10] and casein hydrolysates [11]. The calcium-binding activity of peptides was affected by the molecular weight and the amino acid composition of the peptide [7]. Additionally, the chelation temperature, time, pH, and mass ratio of peptides to calcium also affected the calcium-binding activity of the peptides [8].

Chicken blood is primarily derived from the processing of chicken meat, making it one of the major by-products. A significant volume of chicken blood is produced on a global scale annually, and its predominant utilization is in the form of animal feed conversion. It is rich in proteins and is mainly composed of plasma and hemoglobin. Hemoglobin is the predominant protein with high nutritional value and is abundant in essential amino acids. Hemoglobin has been previously reported as a source of bioactive peptides [12,13]. However, the study of peptide–calcium chelates derived from chicken blood hemoglobin is relatively limited.

Therefore, the purpose of the study was to investigate calcium-binding peptides prepared from chicken blood, optimize the preparation conditions, and investigate the characteristics and stability of the peptide–calcium complexes. This foundation would provide a basis for the development of calcium supplements and functional food materials while improving the utilization value of poultry blood.

## 2. Materials and Methods

### 2.1. Materials and Chemical Reagents

Fresh chicken blood was provided by Jiangsu Lihua Animal Husbandry Co., Ltd. (Changzhou, China). Alcalase 2.5 L was obtained from Aladdin Co., Ltd. (Beijing, China). Pepsin and pancreatin, used for simulated gastrointestinal digestion, were purchased from Hefei Bomei Biotechnology Co., Ltd. (Hefei, China). Ultrafiltration membranes at 5 kDa and 3 kDa of Merck Millipore were purchased from Keli Zehua Technology Co., Ltd. (Beijing, China). All other reagents were of analytical grade and obtained from Jiancheng Chemical Regent Co., Ltd. (Nanjing, China).

### 2.2. Preparation of Blood Peptides

Chicken hemoglobin was extracted using the method described by Hu et al. [14], and the method of enzymatic hydrolysis was prepared using the modified method of Yang et al. [15]. Sodium citrate (3 g/L blood) was quickly added to fresh chicken blood and stirred well to prevent clotting. The blood was centrifuged at 3000× *g* at 4 °C for 15 min to obtain the blood cells. The cells were washed with an equal volume of sterilized saline. This procedure was repeated three times. The cells were frozen, thawed, homogenized in deionized water, filtered, and centrifuged to obtain hemoglobin. It was hydrolyzed with alcalase (1:1000 *w*/*v*) at 50 °C for 8 h and heated in a 95 °C water bath to stop the hydrolysis. The mixture was then filtered using 5 kDa and 3 kDa ultrafiltration membranes. The filtrate was lyophilized and was stored at −20 °C. The sample was considered chicken hemoglobin peptides (CPs).

### 2.3. Chelation Reaction and the Optimization

The calcium-chelating peptides of chicken hemoglobin were prepared according to a modified method described by Wu et al. [16]. Four main factors (mass ratio, pH, temperature, and time) were selected for single-factor experiments based on those that affected the progress of a chemical reaction [17]. The CP samples were dissolved in deionized water, and then CaCl_2_ was added with different mass ratios (2/1 to 10/1). The mixture was incubated at different temperatures (30–70 °C) and pH (5–9) for 20 to 100 min in a water bath. Following the chelation reaction, 9 times the volume of absolute ethanol was added into the reagent to separate the chelate. Subsequently, the mixture was centrifuged at 10,000× *g* for 10 min. The supernatant was collected to calculate the calcium-chelating capacity. The precipitate was collected, and the content of calcium was detected using flame atomic absorption spectrometry. Calcium-chelating capacity was calculated according to the following formula:

(1) $$\mathrm{Calcium}\;\mathrm{chelating}\;\mathrm{capacity}=\frac{M_{T}-M_{U}}{M_{T}}$$

where M_T_ is the calcium content added to the solution (mg), and M_U_ is the calcium content in the supernatant after centrifugation (mg).

According to the single-factor test, a three-level, four-factor BBD central composite design was used to determine the optimal conditions for the preparation of peptide–calcium chelate. The chelate prepared under the optimal conditions was marked as CP-Ca for further analysis.

### 2.4. Analysis of Amino Acids

The amino acid compositions of CP and CP-Ca were tested according to the method by Yang et al. with a Hitachi L-8900A auto amino acid analyzer (Hitachi Ltd., Tokyo, Japan) [18].

### 2.5. Characterization of CP-Ca

#### 2.5.1. Ultraviolet–Visible (UV) Spectroscopy Analysis

The CP and CP-Ca samples were characterized by ultraviolet–visible spectroscopy analysis in the wavelength range 190–400 nm using a UV spectrophotometer (Precision Instrument Co., Ltd., Shanghai, China). For detection, CP and CP-Ca were dissolved in deionized water (50 μg/mL), and deionized water was used as a blank. The scanning interval was 1 nm, and the path length of the quartz cuvette was 1 cm.

#### 2.5.2. Fluorescence Spectroscopy Analysis

The fluorescence spectra of CP and CP-Ca were recorded using an FL-4600 fluorescence spectrophotometer (LS-55, Perkin Elimer Co., Ltd., Llantrisant, UK). The detection conditions were as follows: emission wavelength, 295–500 nm; excitation wavelength, 280 nm; scanning speed, 300 nm/min; and step size, 2 nm.

#### 2.5.3. Fourier-Transform Infrared Spectroscopy (FTIR)

CP and CP-Ca were loaded onto an FTIR instrument (intelligent Nicolet iS50, Thermo Fisher Scientific Co., Ltd., Waltham, MA, USA) for scanning. The scanning range was 525–4000 cm^−1^, and the spectra were acquired using 32 scans.

#### 2.5.4. Scanning Electron Microscopy (SEM)

The surface morphologies of the CP and CP-Ca were examined using a scanning electron microscope (EVO-LS10, ZEISS, Jena, Germany). The lyophilized samples were fixed on the sample plate and sprayed with a gold plating film under high-vacuum conditions. The morphology was observed by SEM at 1500× and 10,000× magnification.

#### 2.5.5. X-ray Diffraction (XRD) Analysis

A D2 PHASER ZX-28 X-ray diffractometer (Bruker, Saarbrucken, Germany) was used to analyze the crystal structures of the CP and CP-Ca. The test conditions were as follows: Anode, Cu; filter nickel tube voltage, 40 kV; acceleration current, 40 mA; scanning speed, 0.02°/0.2 s; and scanning range, 5–80°.

#### 2.5.6. Thermogravimetry and Differential Scanning Calorimetry (TG-DSC)

The thermal properties of the CP and CP-Ca were determined using TG-DSC (TG/DTA7200, Waters, Milford, CT, USA). The sample powder was sealed in a crucible plate and heated from 25 °C to 800 °C at 10 °C/min under a nitrogen atmosphere at 50 mL/min.

### 2.6. In Vitro Stability of Chelates

#### 2.6.1. Stability of CP-Ca at Different pH and Temperatures

The stability of CP-Ca at different pH values and temperatures was evaluated according to the method described by Vo et al. [19]. The CP-Ca sample was dissolved (2 mg/mL) and incubated at 37 °C before the treatment at various pH (2–9) or temperatures (30–80 °C) for 1 h. Subsequently, the solution was centrifuged at 8000 rpm for 10 min for the supernatant. The stability of CP-Ca was expressed as the retention rate, which was calculated as follows:

(2) $$\mathrm{Calcium}\;\mathrm{retention}\;\mathrm{rate}\;\%=\frac{A_{0}-A_{1}}{A_{0}}\times 100$$

where A_0_ represents the calcium content in the sample (mg) and A_1_ represents the Ca content in the supernatant (mg).

#### 2.6.2. Gastrointestinal Stability of CP-Ca In Vitro

The calcium solubility under simulated gastrointestinal digestion was determined based on the method described by Hu et al. with modifications [20]. For the simulated gastric stage, samples were digested with 40 mg of porcine pepsin in 0.1 M HCl (pH 2) and incubated at 37 °C for 2 h. Enzyme activity was terminated by heating at 100 °C for 10 min. For the simulated intestinal stage, samples were digested with 20 mg trypsin in 0.1 M NaHCO_3_ (pH 7.6) for another 2 h, and the digestion was terminated by heating, as described above. The simulated gastrointestinal digestion was conducted as follows: the pH of the solution after the simulated gastric stage was adjusted to 7.6, and then trypsin dissolved in 0.1 M NaHCO_3_ was immediately added to the mixture for 2 h, and then the digestion was terminated by heating. The solution was centrifuged, and the calcium content of the supernatant was determined to calculate the calcium ion stability of CP-Ca *in vitro*. The stability of CP-Ca in simulated gastrointestinal digestion was determined by Formula (2) above.

### 2.7. Statistical Analysis

Data are expressed as mean ± standard deviation. Response surface data were analyzed using Design Expert 12.0, and other data were analyzed by one-way analysis of variance (ANOVA) using Origin (Origin, 2022).

## 3. Results

### 3.1. Calcium-Chelating Rate of CP

**Figure 1:**
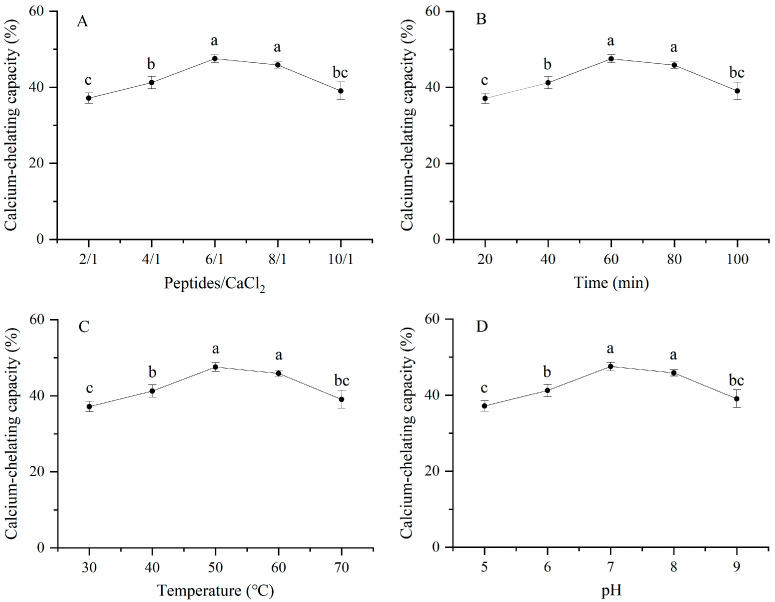
Effects of mass ratio of peptides/CaCl_2_ for (**A**), time (**B**), temperature (**C**), and pH (**D**) on calcium-binding capacity. The different letters mean the significant difference within the groups (*p* < 0.05).

The CP-Ca was prepared by the optimization experiment with a calcium-chelating capacity. The single-factor experimental results are shown in Figure 1. The calcium-chelating capacity increased gradually as the ratio of peptides/CaCl_2_ increased from 2/1 to 6/1 (Figure 1A). As the ratio increased further, the chelating rate decreased. This may be due to excess peptides in the solution, resulting in less collision between the calcium ions and binding sites. Additionally, excess peptides lead to competition for the limited binding sites, which could result in a decrease in Ca^2+^ combined with the per unit mass of CP. There was no significant difference in the chelating capacity when the ratio of peptides/CaCl_2_ increased from 6/1 to 8/1. However, the chelate yield increased. Therefore, the 1/8 peptides/CaCl_2_ ratio was selected as the central point.

The calcium-chelating capacity increased from 20 to 60 min, followed by a decrease (Figure 1B), indicating that this reaction was rapid. This is similar to the results obtained for whey peptides [7]. There was no significant difference in the calcium-chelating capacity between 60 and 80 min. Considering efficiency and energy consumption, 60 min was the center point of the response surface experiment. Figure 1C shows that the calcium-chelating capacity increased significantly as the temperature increased from 30 °C to 50 °C. However, the chelating rate did not change significantly when the temperature was further increased to 60 °C. When the temperature continued to increase above 60 °C, chelating activity decreased significantly. This was similar to the effect of temperature on the calcium-chelating rate of pig bone collagen peptides [16], which also showed a trend of first increasing and then decreasing. Wang et al. also observed the same phenomenon [21]. This may be because the reaction requires a higher temperature to enhance the probability of collision between the calcium and peptides, and temperature could affect the reaction rate and equilibrium coefficient of the calcium peptide complex [22].

As shown in Figure 1D, the calcium-chelating capacity first increased and then decreased with an increase in pH. When the pH was 7 or 8, the difference in the chelating rate was not significant. As the pH increased from 5 to 8, the improvement in the calcium chelating capacity might have been due to the coordination between Ca^2+^, the carboxyl group (COOH−), and ammonia ions (NH3+) [16]. When the pH increased above eight, the calcium-chelating capacity decreased. The reason for the decrease may be that hydroxide (–OH) can form hydroxide precipitates in the reaction solution, which affects the generation of CP-Ca [23]. Therefore, the central points obtained by the single factor test were 60 min, 1/8 peptides/CaCl_2_, 50 °C, and pH 8.

### 3.2. Optimization of Calcium-Binding Conditions of CP

The chelating assay was further optimized based on a single-factor test. The design and results obtained using Design-Expert 12.0 Design software are presented in Table 1. The quadratic regression equation showed the calcium-chelating capacity as a function of temperature (A), the mass ratio of the peptide/calcium (B), chelating time (C), and pH (D).

(3) $$Y\;=76.20+0.8675A\;-0.0428B\;+0.3972C\;-0.4647D\;-0.98\mathrm{AB}\;+2.47\mathrm{AC}\;+3.48\mathrm{AD}\;+0.38\mathrm{BC}\;\;+0.664\mathrm{BD}\;+0.558\mathrm{CD}\;-5.32A^{2}-1.23B^{2}-2.14C^{2}-2.33D^{2}$$

An analysis of variance was used to evaluate the suitability and statistical significance of the quadratic model. As shown in Table 2, the model selected by the test was highly significant (*p* < 0.0001). The *p*-value of the lack of fit was 0.2645, which indicated that the lack of fit was not significant. The model was reliable and could fit the experimental results and adequately account for the regression response relationship. The results showed that AC, AD,C^2^, and D^2^ had a significant effect on calcium-binding capacity (*p* < 0.01).

Based on the model, the optimal chelation parameters were recommended as follows: temperature, 48.47 °C; mass ratio of peptides/CaCl_2,_ 7.98/1; time, 60.48 min; pH, 7.8; and the highest calcium-chelating capacity was predicted to be 77.49%. The accuracy of the model was verified by a validation test. Taking the actual operation of the experiment, the predicted parameters were adjusted as follows: chelating temperature, 50 °C; mass ratio of peptides/CaCl_2_, 8/1; chelating time, 60 min; and pH 7.8. The test was repeated three times under these conditions, and the actual calcium-chelating capacity was 77.54% ± 1.65%, which is similar to the predicted value. The results indicate that the model fits with the actual situation. The chelates were prepared under the optimization situation, and the calcium content was detected. It was found that the calcium content of CP-Ca was 73.27 mg/g. For the research of sheep bone peptides, Atlantic salmon bone peptides and pig bone peptides reported that the calcium-chelating capacities under the optimal calcium-chelating conditions were 42.57%, 52.47%, and 71.93%, respectively [8,21,24]. The chelating capacities were lower than those of CP. The calcium content of the calcium peptide complex of mung beans and Atlantic salmon bone peptide were 42.7 mg/g and 53.45 mg/g under the optimal calcium-chelating conditions, respectively, [17,25], and was also lower than that of CP-Ca. These results indicate that CP-Ca is an excellent source of peptide–calcium chelates.

### 3.3. Analysis of Amino Acids

**Figure 2:**
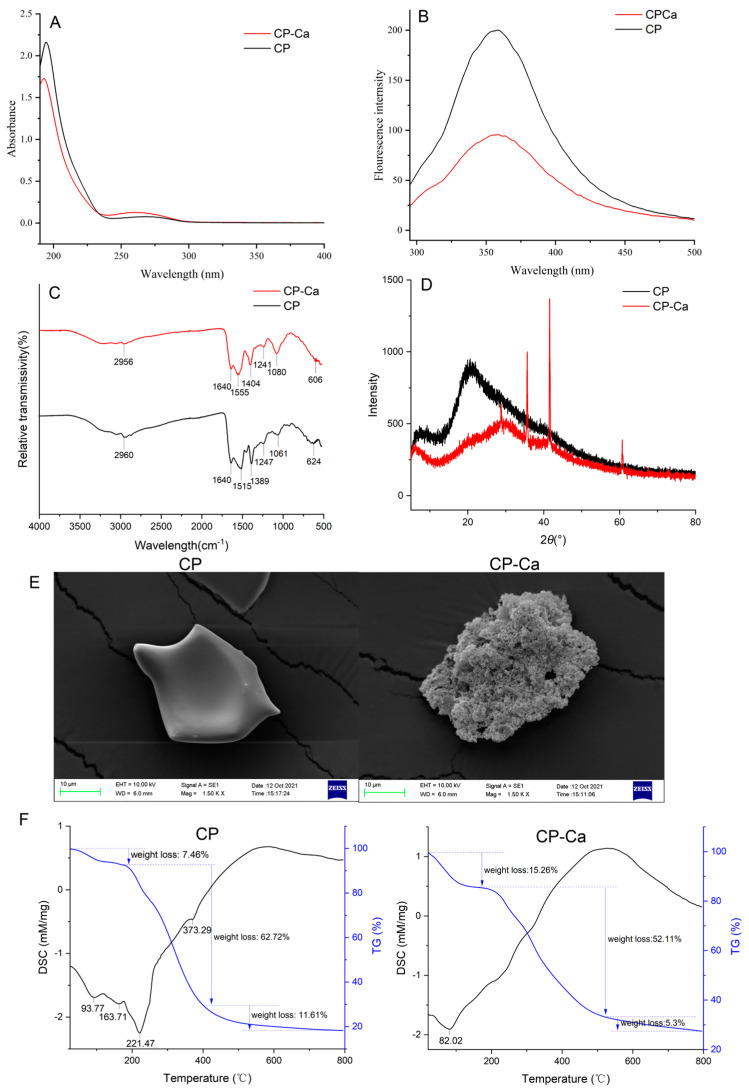
The characterization of CP-Ca. (**A**) Ultraviolet spectra of CP and CP-Ca. (**B**) Fluorescence spectra of CP and CP-Ca. (**C**) Fourier-transform infrared (FT-IR) spectra of CP and CP-Ca in the region from 4000 to 500 cm^−1^. (**D**) X-ray diffraction pattern of CP and CP-Ca. (**E**) Scanning electron microscope images of CP and CP-Ca at ×1500 magnification. (**F**) Typical TG-DSC thermograms of CP and CP-Ca.

Current reports show that amino acid composition and arrangement have an effect on calcium-chelating capability [26,27]. As shown in Table 3, the amino acids of CP and CP-Ca are similar. However, the relative content of amino acids was significantly changed. The total amino acid content was reduced from 88.13 g/100 g to 61.79 g/100 g. The decrease may be due to the increase in moisture content (mass change from 7.46% to 15.26% in Figure 2F) and the occupation of calcium ions (73 mg/g) in the chelate. In addition, not all the peptides were chelated, and some peptides in the supernatant were not included in the chelate. Both CP and CP-Ca contained all kinds of amino acids. The essential amino acid content of CP and CP-Ca was 42.35% and 28.36%, respectively. It is known that an adequate intake of essential amino acids stimulates the body to produce serotonin, glutathione, and other substances, which benefits health [28]. The nutritional value of protein depends mainly on essential amino acids. The proportion and content of essential amino acids are closer to the FAO/WHO model, and the nutritional value of protein is better [29]. Thus, both had high nutritional value. In addition, CP was rich in glutamic acid (Glu), lysine (Lys), leucine (Leu), alanine (Ala), and aspartic acid (Asp), whereas CP-Ca was rich in Glu, Asp, Lys, and Ala. Compared with CP, the content of negatively charged amino acids (Glu and Asp) in CP-Ca increased significantly and reached 43%. Many studies reported that Glu and Asp were the most abundant amino acids in the calcium-chelating peptide [7,8,21,22,23]. The results indicate that these amino acids may promote the chelation of peptides and Ca^2+^. This may be due to the important contribution of the carboxyl groups of Glu and Asp to their chelation [30,31].

### 3.4. Characterization Results of CP and CP-Ca

#### 3.4.1. UV Spectroscopy Analysis

The variation between peptides and peptide–calcium chelates can be detected through ultraviolet absorption intensity and dislocation [32]. As shown in Figure 2A, a strong absorption peak was observed at 195 nm. The peak of the peptides chelated with calcium moved to 193 nm, and the absorption intensity decreased from 2.16 to 1.73, which showed a hypochromic effect and blue shift phenomenon. In addition, both CP and CP-Ca had an absorption peak at approximately 260 nm, although the shape and intensity were different. The chelation of chromophore groups (-COOH, -C=O) or auxochrome groups (-NH_2_, -OH) in the peptides and the metal ion may result in the displacement or disappearance of the original absorption peaks or the appearance of new absorption peaks [31]. The chromophore groups of the peptides were the carbonyl (-C=O), -COOH, and amide group (-CO-NH-), and the auxochrome groups of the sample were the -OH and an amino group (-NH_2_). Figure 2A shows a clear difference between the UV spectra of CP and CP-Ca. This indicated that the peptides chelated with Ca^2+^ and suggested that some chromophore groups or auxochrome groups might be involved in the chelating reaction.

#### 3.4.2. Fluorescence Spectroscopy Analysis

The fluorescence spectra of CP and CP-Ca are shown in Figure 2B. Both show a maximum fluorescence emission at approximately 358 nm when excited at 280 nm. However, the fluorescence intensity decreased significantly when chelated with calcium, similar to that reported by Zhou et al. [33]. Aromatic amino acids can emit fluorescence, which can be determined using fluorescence spectroscopy [31]. Figure 2B shows that the fluorescence intensity of the chelates was lower than that of peptides, and Table 3 shows that the content of aromatic amino acids (Tyr and Phe) in the chelates was lower than that of the peptides. These results are consistent. The fluorescence quenching phenomenon indicates that folding and aggregation may be induced when CP is chelated with Ca^2+^. The same fluorescence quenching was also observed in peptide–calcium chelates from fish bone [34].

#### 3.4.3. FTIR Analysis

FTIR can reflect the interactions between metal ions and organic functional groups [21]. Therefore, it is used to indicate the change in the organic ligand groups in the peptides. The FTIR spectra of CP and CP-Ca are shown in Figure 2C. The peaks around 1640 cm^−1^ are characteristic of amide I (-C=O), which were observed in both CP and CP-Ca. When CP was chelated with calcium ions, a new peak was observed at 1241 cm^−1^, which may be due to the stretching of C-N. The characteristic wavenumber of the N-H group is 1515 cm^−1^. In the chelates, it changed to 1555 cm^−1^. This indicates that chelation causes the stretching of the N-H group. Similarly, the wavenumber shifted from 1389 cm^−1^ to 1404 cm^−1^, which might be caused by -COOH. The shift in the band at 1061 cm^−1^ to a deeper peak at 1080 cm^−1^ corresponds to the vibration of the C-O-C bonds. In the wavenumber of 500–1000 cm^−1^, several absorption peaks changed after chelation with Ca^2+^, which may be caused by the vibrations of the C-H and N-H bonds [35,36]. These data were similar to those obtained for calcium chelation by chicken foot protein hydrolysate [35], *Auxis thazard* peptides [36], and wheat germ protein hydrolysates [37]. The chelation of CP and Ca^2+^ resulted in different FTIR spectra for CP and CP-Ca. In summary, it infers that carbonyl oxygen, carboxyl oxygen, and amino nitrogen were involved in chelation. These results are similar to those of pig bone peptides [16] and chicken foot peptides [35].

#### 3.4.4. XRD Analysis

XRD is a typical method to evaluate changes in crystalline structure. Figure 2D clearly shows differences in XRD patterns after CP chelated with Ca^2+^. The peak of CP was around 20.6°, but its intensity was weak, and the area was large, illustrating that the structure of CP was irregular amorphous. This is consistent with the XRD of tilapia bone peptides [38].

However, high intensity and sharp absorption peaks were observed in CP-Ca. These peaks were generated mainly due to the coordinate bond between the CP and Ca^2+^ [39]. In addition, after the chelation reaction, the peak at 20.6° disappeared, and small but sharp absorption peaks appeared. These changes indicate that CP and CaCl_2_ formed new crystal structures [40], but the chelation was not mechanically mixed [41].

#### 3.4.5. SEM Results

The microstructures of CP and CP-Ca were observed by SEM (Figure 2E). CP-Ca was identified as different from CP. CP presented a smooth, homogeneous plate structure; however, CP-Ca was compact and granular. Additionally, the size of CP-Ca was larger than CP. These results are similar to those reported for tilapia bone peptides [22]. This difference may be due to the destruction of the original structure by the interaction between peptides and metal ions [42], and the granular structure may be formed through ligand binding, ion binding, and adsorption [36].

#### 3.4.6. TG-DSC Analysis

The TG-DSC curves are shown in Figure 2F. The TG curves were similar, and weight loss progression for both CP and CP-Ca was divided into three stages. The weight loss in CP and CP-Ca was 81.83% and 72.67%, respectively. In the first stage, the mass loss was caused by the volatilization of water in the sample. In the second stage, CP lost 62.72% of weight from 175 °C to 400 °C, and CP-Ca lost 52.11% of weight from 195 °C to 535 °C. Both the starting and ending temperatures of CP-Ca were higher than those of CP, indicating that CP-Ca is more thermostable than CP. The temperatures of endothermic peaks were found at 93.77 °C, 163.71 °C, 221.47 °C, and 373.29 °C through the DSC analysis. These peaks were mainly caused by the destruction of C-N bonds [43]. With the exception of the first endothermic peak of CP-Ca, no clear endothermic peak was found. The results indicate that the bond of CP-Ca required more heat to be broken, which is consistent with the TG curves. These results show that the thermal stability of CP-Ca was higher than that of CP, indicating that CP-Ca may be used as a functional food and medicinal ingredient.

### 3.5. Stability Analysis of Chelates In Vitro

**Figure 3:**
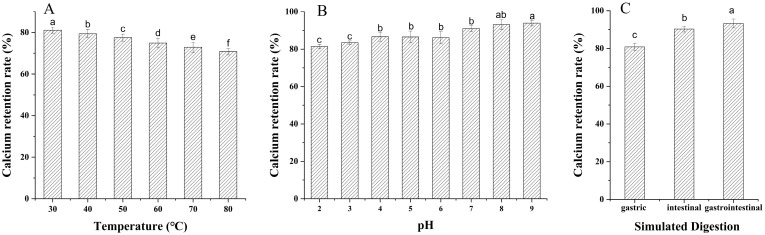
Stability of CP-Ca. (**A**) pH stability. (**B**) Temperature stability. (**C**) Simulated digestion *in vitro*. Different lowercase letters at the top of the bars indicate a significant difference in values among samples (*p* < 0.05).

The bioavailability of calcium ions is affected by temperature, pH, and the human gastrointestinal environment [3]. As shown in Figure 3A, the calcium retention rate of CP-Ca slightly decreased with increasing temperature. When heated to 80 °C, it was still above 70%. These results indicate that CP-Ca was stable at 30–80 °C. The calcium retention rate decreased from 93.91% to 81.39%, whereas the pH ranged from 9 to 2 (Figure 3B). When CP-Ca was in a neutral or alkaline environment, it exhibited high stability. This may be because CP-Ca decomposed, and calcium ions competed for the chelating sites with hydrogen ions. The calcium retention rate in the acidic solutions was lower than that in the alkaline solution (Figure 3B). These results are similar to those of iron-chelating peptides from cattle bone collagen [42]. This indicates that CP-Ca is relatively stable over a wide range of pH values and may be used for functional foods prepared under these processing conditions.

It has been reported that peptides may be hydrolyzed in the gastrointestinal tract, reducing their biological activity. Therefore, the calcium retention rate of the CP-Ca was tested to evaluate digestive stability in the gastrointestinal environment, and the results are shown in Figure 3C. The calcium retention rate of the chelates remained at 80.80%, 90.23%, and 93.24% under simulated gastric digestion, intestinal digestion, and gastrointestinal digestion, respectively. Although digestion decreased the calcium retention rate, the calcium retention rate of chelates remained extremely high, indicating that CP-Ca was stable in the gastrointestinal environment. The pH value of the gastric digestion solution was lower, and its calcium retention rate was lower, which is consistent with the results shown in Figure 3B. A low pH leads to the chelate complex release of metal ions and reduces its stability [44], whereas a higher pH results in the release of fewer metal ions and more stable chelates [17]. The results show that CP-Ca is stable in the gastrointestinal environment and is suitable for promoting calcium absorption.

## 4. Conclusions

In this study, the chelation process parameters were optimized, and the structural characterization and stability of the CP-Ca were studied. The calcium-binding ability was 77.54% after the optimization test. The physical characteristics of CP-Ca were clearly different from CP. The spectral analysis indicated that the chelating sites of CP-Ca were carbonyl oxygen, carboxyl oxygen, and amino nitrogen. The microstructure results showed that the chelating leads to structural change. The TG-DSC and stability analysis indicated that the chelates exhibited excellent thermostability and good stability in the acid-base and simulated digestion solutions. Therefore, CP-Ca may be developed as an enhancer of Ca absorption in functional foods or calcium products. However, further research on their structures and absorption mechanisms in vivo should be conducted.

## Figures and Tables

**Table 1:** Response surface design and results.

Std	Run	A (°C)	B (m/m)	C (min)	D: pH	Calcium-Binding Capacity (%)
27	1	50	8	60	8	77.7269
28	2	50	8	60	8	76.6701
4	3	55	9	60	8	70.2972
25	4	50	8	60	8	75.9712
7	5	50	8	50	9	70.8109
23	6	50	7	60	9	70.4007
15	7	50	7	70	8	72.9284
2	8	55	7	60	8	71.7431
26	9	50	8	60	8	75.7318
20	10	55	8	70	8	72.6667
9	11	45	8	60	7	72.5602
12	12	55	8	60	9	71.0987
21	13	50	7	60	7	74.0386
11	14	45	8	60	9	63.5485
14	15	50	9	50	8	71.5658
13	16	50	7	50	8	74.2934
17	17	45	8	50	8	70.0932
16	18	50	9	70	8	71.7201
22	19	50	9	60	7	73.9057
24	20	50	9	60	9	67.9245
6	21	50	8	70	7	71.6051
29	22	50	8	60	8	74.9225
1	23	45	7	60	8	67.1004
8	24	50	8	70	9	74.2846
3	25	45	9	60	8	69.5771
10	26	55	8	60	7	66.1717
18	27	55	8	50	8	67.1019
19	28	45	8	70	8	65.7898
5	29	50	8	50	7	70.3631

**Table 2:** Analysis of variance for the quadratic regression model.

Source	Sum of Squares	df	Mean Square	F-Value	*p*-Value	Significant
Model	295.54	14	21.11	11.09	<0.0001	**
A-temperature	9.03	1	9.03	4.74	0.047	*
B-ratio (m/m)	0.022	1	0.022	0.0116	0.9159	
C-time	1.89	1	1.89	0.9946	0.3355	
D-pH	2.59	1	2.59	1.36	0.2628	
AB	3.85	1	3.85	2.02	0.177	
AC	24.35	1	24.35	12.79	0.003	**
AD	48.57	1	48.57	25.52	0.0002	**
BC	0.577	1	0.577	0.3031	0.5906	
BD	1.76	1	1.76	0.927	0.352	
CD	1.25	1	1.25	0.6541	0.4322	
A^2^	183.92	1	183.92	96.62	<0.0001	**
B^2^	9.84	1	9.84	5.17	0.0393	*
C^2^	29.72	1	29.72	15.61	0.0014	**
D^2^	35.2	1	35.2	18.49	0.0007	**
Residual	26.65	14	1.9			
Lack of Fit	22.19	10	2.22	1.99	0.2645	
Pure Error	4.46	4	1.11			
Cor Total	322.18	28				
R^2^	0.9133			Adjussted R^2^	0.8267	
Adeq Precision	12.3298			Predicted R^2^	0.8589	

** Significant within a 99.9% confidence interval. * Significant within a 95% confidence interval.

**Table 3:** Amino acid composition of CP and CP-Ca.

Amino Acids	CP (%)	CP-Ca (%)	Amino Acids	CP (%)	CP-Ca (%)
Asp ^#^	9.29 ± 0.34	19.92 ± 0.79	Ile *	3.53 ± 0.07	3.09 ± 0.35
Thr *	5.67 ± 0.22	4.31 ± 0.28	Leu *	9.57 ± 0.11	4.87 ± 0.39
Ser	4.20 ± 0.27	3.44 ± 0.16	Tyr	3.51 ± 0.04	2.47 ± 1.02
Glu ^#^	12.12 ± 0.42	23.09 ± 1.03	Phe *	4.98 ± 0.23	2.87 ± 0.45
Gly	4.33 ± 0.34	4.55 ± 0.27	Lys *	10.04 ± 0.18	8.25 ± 0.56
Ala	9.30 ± 0.97	6.52 ± 0.48	His	5.47 ± 0.06	4.84 ± 0.43
Cys	1.50 ± 0.41	1.38 ± 0.35	Arg	5.32 ± 0.58	3.07 ± 0.54
Val *	6.71 ± 0.20	4.26 ± 0.53	Pro	2.61 ± 0.08	2.36 ± 0.27
Met *	1.86 ± 0.12	0.71 ± 0.19	Total	100	100

^#^ negatively charged amino acids; * essential amino acids.

## Data Availability

The original contributions presented in the study are included in the article, further inquiries can be directed to the corresponding authors.
